# Purification and Chemical Characterization of a Potent Acaricide and a Closely Related Inactive Metabolite Produced by *Eurotium rubrum* C47

**DOI:** 10.3390/antibiotics9120881

**Published:** 2020-12-09

**Authors:** José F. Ortiz-Lemus, Sonia Campoy, Librada M. Cañedo, Paloma Liras, Juan F. Martín

**Affiliations:** 1 Instituto de Biotecnología de León (INBIOTEC), Parque Científico de León, Av. Real, 1, 24006 León, Spain; josefelix@unipamplona.edu.co (J.F.O.-L.); scampoy@biosearchlife.com (S.C.); paloma.liras@unileon.es (P.L.); 2 Área de Microbiología, Departamento de Biología Molecular, Universidad de León, 24071 León, Spain; 3 Departamento de Microbiología, Universidad de Pamplona, Pamplona 543050, Colombia; 4 Research and Development Department, PharmaMar S.A., 28770 Madrid, Spain; lcanedo@pharmamar.com

**Keywords:** mites, *Eurotium*, biological control, flavoglaucin, aspergin, cured ham, cecina

## Abstract

Mites are arthropods and some of them infest dry meat cured products and produce allergic reactions. Some mites, such as *Tyrolichus casei*, *Tyrophagus putrescentiae,* or *Tyrophagus longior* feed on filamentous fungi that grow during the meat curing process. Removal of mite infestation of meat products is extremely difficult and there are no adequate miticidal compounds. The filamentous fungus *Eurotium rubrum* growing on the surface of ham is able to exert a biocontrol of the population of mites due to the production of miticidal compound(s). We have purified two compounds by silica gel chromatography, gel filtration, semipreparative and analytical HPLC and determined their miticidal activity against *T. casei* using a mite feeding assay. Mass spectrometry and NMR analysis showed that these two compounds are prenylated salicilyl aldehydes with a C-7 alkyl chain differing in a double bond in the C-7 alkyl chain. Structures correspond to those of flavoglaucin and aspergin. Pure flavoglaucin has a miticidal activity resulting in more than 90% mite mortality whereas aspergin does not affect the mites. Both compounds were formed simultaneously by *E. rubrum* C47 cultures in different media suggesting that they are synthesized by the same pathway. Production of both compounds was higher in solid culture media and the products were associated with abundant formation of cleistothecia. In liquid cultures both compounds remained mainly cell-associated and only about 10% of the total compounds was released to the culture broth. This miticidal compound may be used to combat efficiently mite infestation in different habitats. These results, will promote further advances on the utilization of flavoglaucin in food preservation and in human health since this compound has antitumor activity.

## 1. Introduction

Mites are small arthropods that belong to the subclass Acari. Although 30,000 species of mites have been described probably the number of species is even larger [1]. Mites are ubiquitous organisms that grow in a large variety of habitats, particularly in soil, the skin of animals, meat, and meat-derived dry cured products, dry fruits [2], grains, and stored food [3] and on the surface of cheese [4,5] and house-hold carpets and bedclothes. Mites may infest honeybees and this affects the reproduction of these insects causing an important economic problem [6,7] Some mites transmit diseases to human and domestic animals and produce severe allergic reactions including asthma, rhinitis, and dermatitis [8,9,10]; therefore, their control is an important economical and medical problem and there is a strong interest in the removal of mite infestations.

Some meat products of high quality, such as dry ham and dry beef cecina are cured by hanging in cold and dry cellars for several months, allowing the growth on their surfaces of a lawn of filamentous fungi that contribute to the maturation process [11,12]. Occasionally, these meat products are infested with mites during the curing process, especially when the temperature is 15 °C and the humidity is above 70%.

Some mites are fungivors feeding on spores and mycelium of filamentous fungi [13,14,15] as is the case of mites belonging to genus *Tyrophagus* and *Tyrolichus*, that proliferate and damage the surface of cured ham and cecina [16]. Efforts have been dedicated to the search of suitable acaricidal compounds to combat the mite infestations [17]. Classical chemical acaricidal compounds cannot be used to remove mites from these cured meat products used for human consumption.

The population of mites of the *Tyrophagus* and *Tyrolichus* genus is high when the meat products are covered with species of *Penicillium* and some *Aspergillus* species, whereas this population is reduced when the surface of the ham is covered by xerophilic *Eurotium* species. Therefore, we hypothesized that the lower population of mites in *Eurotium* covered ham was due to an antagonistic effect exerted on the growth of the mites by some bioactive compounds produced by certain *Eurotium* species [18]. We selected a strain, *Eurotium rubrum* C47, that exerted a strong inhibitory effect on the growth of *T. casei*, *T. putrescentiae,* and *T. longior* at all stages of the mite development, from egg deposition to nymph and adult mites [18]. Therefore, it was of interest to isolate in pure form the bioactive miticidal compound(s) produced by *E. rubrum* C47 to characterize the chemical structure of the toxic molecules and to study its secretion process. In this article we report the purification process of the miticidal compound by silica-gel chromatography, as well as preparative and analytical HPLC. The structure of two biosynthetically related compounds has been established by MS and NMR; we found that one of them was drastically more active than the other in the control of mites. The production of these compounds has been optimized in several culture media, and we observed that both compounds were produced at distinct levels in the cultures of different strains, and remained largely cell-associated.

## 2. Results

### 2.1. Purification of the Acaricidal Compound

In previous studies we observed that the production of an acaricidal compound was higher in cultures of *E. rubrum* C47 grown for 7 to 14 days in plates of solid CY20SM medium [18]. Therefore, mycelium of this fungi was collected from the surface of 40 CY20SM plates (24 g) and extracted four times with ethyl acetate obtaining an intensively red solution with an acaricidal activity resulting in about 75% mite mortality. The solvent was evaporated to dryness, the solid residue was dissolved in dichloromethane-methanol (4:1 *v*/*v*) and the bioactive extract was loaded into a Silica Gel column equilibrated with dichloromethane. The sample was fractionated by eluting successively with three solvents: Dichloromethane (100%) which produced a yellow-orange eluate (fraction I), dichloromethane-methanol (1:1 *v*/*v*) giving a red eluate (fraction II) and finally dichloromethane-methanol (1:9 *v*/*v*) that originated a purple eluate (fraction III). The three fractions were vacuum evaporated to eliminate the solvent, dissolved in ethanol and their acaricidal activity was evaluated using mite food (DYS) supplemented separately with each fraction and with ethanol as negative control. As shown in Supplementary Materials Figure S1A the mite population growth in cultures containing food supplemented with fractions II and III was similar to that of the control cultures supplemented with ethanol. However, in the plates supplemented with fraction I the mites avoided food consumption, were unable to move and 85% of the initial mite population died after 13 days.

In a second purification step the active fraction I was applied to a Bio Gel S-X3 column in which hydrophobic molecules of molecular weight lower than 2000 Da are separated by size in gel filtration. The compounds in the gel were eluted with acetone, and the successive fractions were combined as follows: Colorless initial fractions (E1), slightly red fractions (E2), yellow fractions (E3), orange fractions (E4), and the final fractions that were again colorless (E5). The acaricidal activity of the original extract was found in eluate E2 which produced an 85% mortality in the mites, while no acaricidal activity was observed in eluates E1, E3, E4, and E5 (Figure S1B). A scanning UV-visible of eluate E2 showed the presence of two peaks of maximal absorption at 350 and 503 nm.

### 2.2. Resolution of Eleven Compounds by Semipreparative and Analytical HPLC

**Figure 1:**
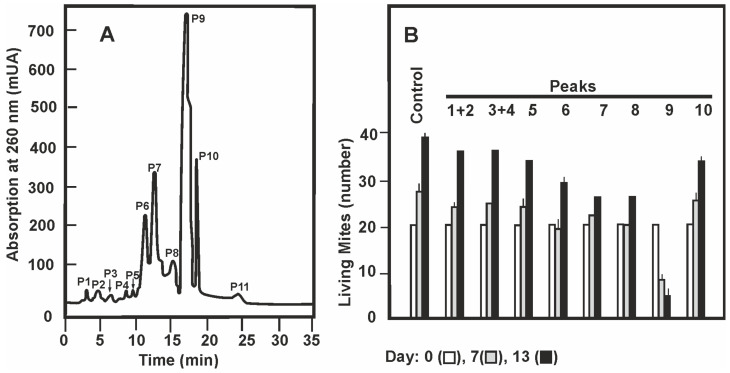
Purification of the miticidal activity by semipreparative HPLC. (**A**) Absorption profile at 260 nm of the fractions eluting from the semipreparative HPLC. The eleven peaks are indicated. (**B**) Acaricidal activity of the peaks obtained in the semipreparative HPLC. Extracts from peaks 1 and 2, and peaks 3 and 4 were mixed and studied jointly. The acaricidal activity was studied along the 13 days of the assay; in the figure the data corresponding to days zero (white bars), 7 (grey bars) and 13 (black bars) are shown. ANOVA analysis of the number of living mites in two replicas for every one of the 9 groups indicates a *p*-value < 0.05 both at 7 and 13 days. The Tukey test shows that the differences of living mite number is significantly different in the sample of peak 9 in relation those of the other peaks.

Thereafter, repeated batches of fraction E2 obtained from the Bio Gel S-X3 gel filtration were applied to a semipreparative HPLC chromatography to obtain sufficient amounts of the active compound. The E2 fraction mixture was resolved by semipreparative HPLC chromatography in eleven compounds absorbing at 260 nm (Figure 1A). Each of these peaks was collected, dried, the solid pellets were dissolved in ethanol and tested for acaricidal activity on *T. casei.* The activity was found only in peak 9, with a retention time of 16.5 min (Figure 1B) while the other peaks did not show acaricidal activity. Therefore, fractions of peak 9 were collected from repeated semipreparative HPLC chromatographies, mixed, lyophilized and further resolved by analytical HPLC.

**Figure 2:**
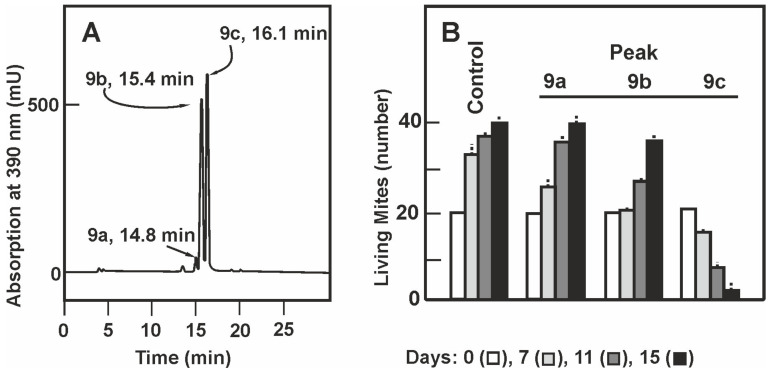
Purification of the miticidal activity by analytical HPLC. (**A**) Absorption profile at 390 nm of the fractions eluting from the analytical HPLC. Peaks 9a, 9b, and 9c are indicated by arrows showing their retention times. (**B**) Acaricidal activity of the peaks obtained in the analytical HPLC. The acaricidal activity was studied along 15 days; only the data corresponding to days zero (white bars), 7 (light grey bars), 11 (dark grey bars), and 15 (black bars) are shown. The data are the average of three experiments and their standard deviations indicated by vertical dashed lines. ANOVA analysis of the number of living mites in the four samples analyzed indicates a *p*-value < 0.05 at all times shown. The Tukey test shows that the differences of living mites in the sample of peak 9c is significantly different from those of the other peaks after 11 days of culture. The differences between samples 9b and 9c were not significant at 7 days of culture.

In analytical HPLC peak 9 was resolved in 3 peaks (Figure 2A) designated 9a, 9b and 9c that eluted with retention times of 14.8, 15.4, and 16.1 min, respectively. Compounds 9a, 9b, and 9c were collected from repeated HPLC analytical chromatographies and each peak was separately tested for acaricidal activity. The population of *T. casei* after feeding with compound 9c avoid feeding in the first few days and after 15 days almost 90% of the population died (Figure 2B). No significant acaricidal activity was found in fraction 9a and very weak activity in fraction 9b although the mites required some time to became adapted and proliferate. Interestingly the compound of fraction 9b showed similar absorption spectrum (Figure S2) as the compound 9c although it lacks significant acaricidal activity.

### 2.3. Monitoring of the Purification of the Active Compound by Thin Layer Chromatography

**Figure 3:**
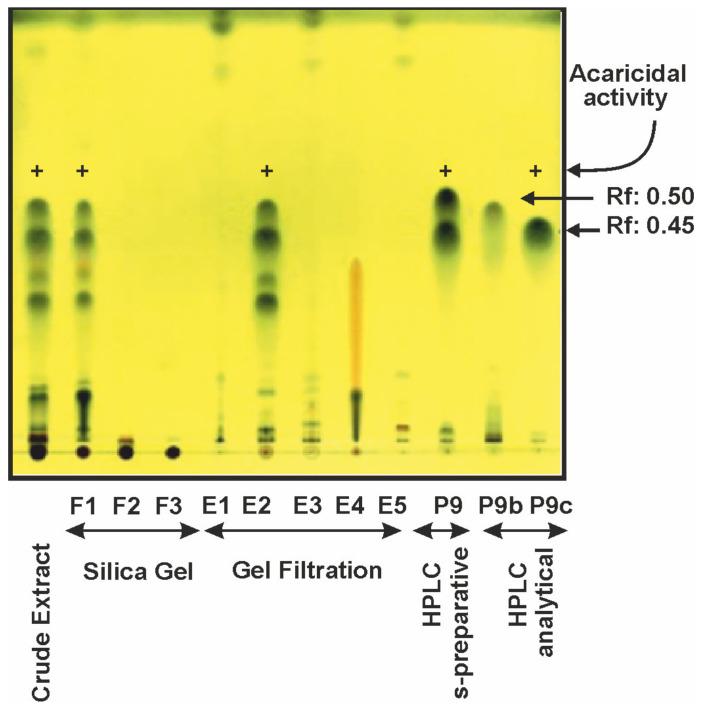
Thin layer chromatography in Silica Gel 60 of extracts from the different steps of purification. The lanes correspond to the crude mycelium extract, the three fractions (F1, F2, and F3) obtained in silica gel chromatography, the five eluates (E1, E2, E3, E4, and E5) obtained in gel filtration chromatography, the biologically active peak 9 obtained in the semipreparative HPLC and the peaks 9b and 9c obtained by analytical chromatography. The fractions with acaricidal activity are indicated above with a (+). The spots indicating peaks 9b (aspergin) and 9c (flavoglaucin) and their Rf are indicated with arrows.

To follow the compound present in the successive purification steps we separated these compounds by thin layer chromatography in all samples obtained from the original crude extract to the analytical HPLC eluted samples. The compounds were detected after staining the chromatosheets with phosphomolibdic acid (Figure 3). An enrichment of compounds 9b and 9c was observed throughout the successive purification steps; the compound 9c, with a Rf of 0.45, was present in all the samples showing acaricidal activity but was absent in samples lacking acaricidal activity.

### 2.4. Structure Determination of Compounds 9b and 9c

Although the acaricidal activity was found in peak 9c, the compounds 9b and 9c shared several properties. Both compounds were soluble in methanol, acetonitrile and chloroform and they showed absorption spectra with peaks at 230, 273, and 390 nm (Figure S2). Compounds 9b and 9c were separated with only 0.7 min of difference in their retention times in analytical HPLC (15.4 and 16.1 min respectively, Figure 2A) and moved closely in TLC (Rf 0.50 and 0.45, Figure 3). The structures of the compounds 9b and 9c were elucidated using 1D and 2D homo- and hetero-nuclear NMR analysis and MS data. The ^1^H and ^13^C NMR data for both compounds are shown in Table 1.

**Figure 4:**
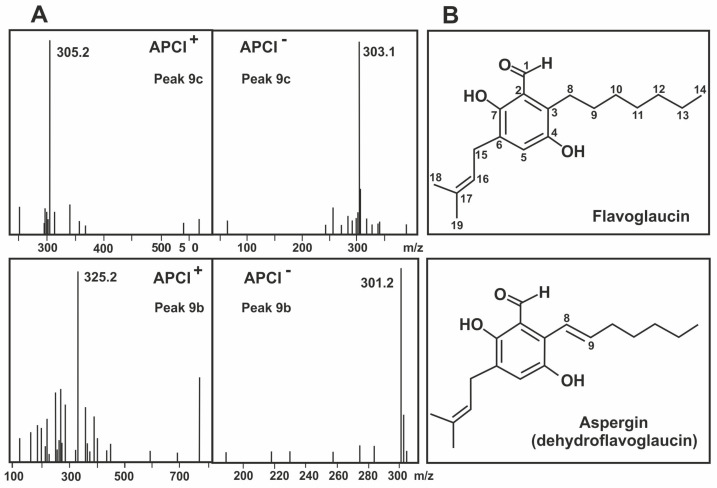
Chemical characterization of the purified compounds. (**A**) Mass spectra, and (**B**) chemical structure of Flavoglaucin (upper panels) and Aspergin (lower panels).

The acaricide compound 9c was isolated as a pale yellow powder, with a molecular formula C_19_H_28_O_3_ deduced from its APCIMS *m*/*z* 305.2 [M + H]^+^ and 303.1 [M − H]^−^ (Figure 4A) as well as its ^13^C NMR data, indicating six degrees of unsaturation. The ^13^C NMR spectrum demonstrated 19 signals which were assigned to six aromatic carbons, two olefinic carbons, three methyls, seven methylenes, and one aldehyde carbonyl by DEPT and HSQC experiments. ^1^H and ^13^C NMR spectra of 9c indicated the presence of one aldehyde group (δ_H_ 10.25 and δ_C_ 195.8), one olefinic proton (δ_H_ 5.58 and δ_C_ 121.4) and one aromatic proton (δ_H_ 6.88 and δ_C_ 125.9) in the structure. Detailed analysis of 2D NMR, COSY, HSQC and HMBC spectra allowed construction of a prenylated benzaldehyde moiety with a heptyl side chain and the precise connectivities of 9c were established by interpretation of HMBC correlations. The compound 9c was identified as flavoglaucin (Figure 4B) on the basis of its spectroscopic data and by comparison with those reported in the literature [19,20].

Compound 9b was also obtained as pale yellow powder, with the molecular formula C_19_H_26_O_3_ as deduced from its APCIMS *m*/*z* 325.2 [M + Na]^+^ and 301.2 [M − H]^−^ measurements (Figure 4A), indicating seven degrees of unsaturation. The ^1^H and ^13^C NMR data of 9b (Table 1) were very similar to those of 9c, the only difference in the ^13^C NMR spectra was that two methylenes (δ_C_ 24.2, C-8 and *δ*_C_ 32.2, C-9) in 9c were replaced by a double bond (δ_C_ 120.3, C-8 and δ_C_ 142.9, C-9) in 9b, according with one additional unsaturation and the position of the double bond was determined by analysis COSY and HMBC data. All the spectroscopic information was compared with the literature and the compound was identified as aspergin (Figure 4B), also named tetrahydroauroglaucin and dehydroflavoglaucin [21,22,23].

### 2.5. Production of Flavoglaucin and Aspergin by E. rubrum C47 in Different Solid Media

**Figure 5:**
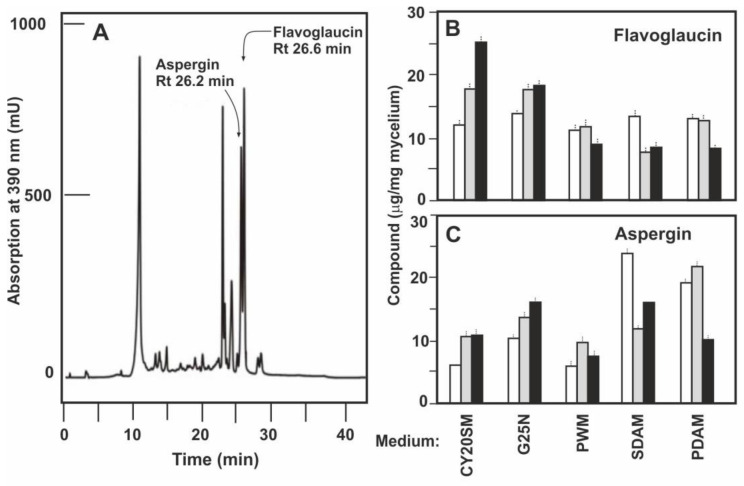
Production of flavoglaucin and aspergin in different solid media. (**A**) HPLC analytic chromatography of an extract of *Eurotium rubrum* C47 mycelium grown in solid CY20SM medium for 14 days. It shows flavoglaucin (Rt 26.6 min) and aspergin (Rt 26.2 min) as demonstrated by co-elution with pure samples of each compound. Production of flavoglaucin (**B**) and aspergin (**C**) by *E. rubrum* C47 grown in the different solid media shown below panel C. The bars correspond to production at 5 days (white bars), 10 days (grey bars) and 14 days (black bars) of culture. Data are the average of three cultures and standard deviations are shown by vertical dashed lines. ANOVA analysis of flavoglaucin and aspergin production in solid media by *E. rubrum* C47 in the three replicas of every five groups indicates *p*-values < 0.05 at the different times analysed. The Tukey test shows that the differences of concentrations of flavoglaucin and aspergin produced in CY20SM medium is significantly different from the production in any of the other media. At 15 days no significant differences in flavoglaucin production were found in SDAM medium in relation to PDAM or PWM media. The same applies to the production of aspergin between G25N and SDAM media.

*E. rubrum* C47 was grown for 14 days in the following solid media: CY20SM, G25N, CY20S, MEAM, PDAM, SDAM, PWM, PW2M, and RTM all of which contained 20% sucrose to decrease the water activity value. Samples were extracted from the mycelium of cultures grown for 5, 10 and 14 days. Production of flavoglaucin and aspergin was measured by analytical HPLC in which flavoglaucin has a retention time of 26.6 min and aspergin shows a retention time of 26.2 min, as determine by co-elution with pure standards of each compound (Figure 5A). Flavoglaucin production was similar in CY20SM and G25N media after 10 days, but after 14 days of culture the largest production was observed in CY20SM medium (25 μg/mg mycelium), being 27% lower in G25N medium. Production in PWM, SDAM and PDAM reached a plateau at 5 days (12 μg/mg mycelium) but then decreased (Figure 5B). The production of flavoglaucin in CY20S medium increased steadily along the culture, up to the order of 10 μg/mg mycelium at 14 days, but in MEAM, RTM, or PW2M media the yield was always below 3 μg/mg mycelium (not shown). Production of aspergin differs from that of flavoglaucin in the distinct media, being maximal in SDAM and PDAM media at 1 day and 5 days of culture, respectively, with values around 20 μg/mg mycelium (Figure 5C). Again, the lowest productions were obtained in CY20S, MEAM, RTM, and PW2M media (not shown).

### 2.6. Production of Flavoglaucin and Aspergin by Different Eurotium Strains

Once the optimal solid media for the production of flavoglaucin was established we studied the production of flavoglaucin and aspergin in CY20SM and G25N media by different *Eurotium* strains. The strains tested were *E. rubrum* C47, previously selected by its high acaricidal activity and the collection culture strains *E. rubrum* NRRL 52, *E. rubruns* NRRL 76, and *E. cristatum* NRRL 4222, all of them showing miticidal activity in previous tests [18]. The production of flavoglaucin and aspergin by the strains *E. repens* NRRL 40, *E. amstelodami* NRRL 90, *E. chevalieri* NRRL 4755, and *E. rubrum* J14a, all of which were consumed by mites without toxicity [18], were tested as negative control.

**Figure 6:**
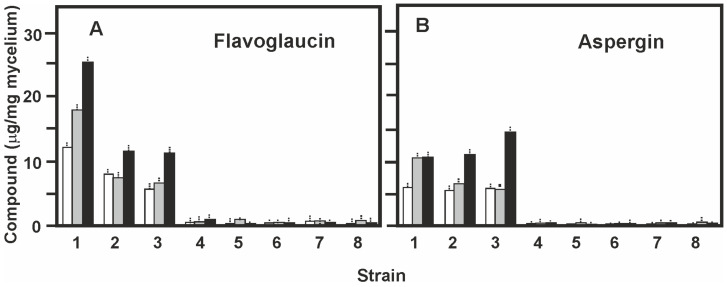
Production of flavoglaucin and aspergin by different *Eurotium* strains. Production of flavoglaucin (**A**) and aspergin (**B**) in solid CY20SM medium by the following strains: *E. rubrum* C47 (1), *E. rubrum* NRRL 52 (2), *E. rubrum* NRRL 76 (3), *E. repens* NRRL 40 (4), *E. amstelodami* NRRL 90 (5) *E. cristatum* NRRL 4222 (6), *E. chevalieri* NRRL 4755 (7), and *E. rubrum* J14a (8). The bars correspond to production at 5 days (white bars), 10 days (grey bars) and 14 days (black bars) of culture. Data are the average of three cultures and the standard deviations are shown by vertical dashed lines. ANOVA analysis of the flavoglaucin and aspergin produced by the eight studied strains in CY20SM medium gives a *p*-value < 0.05. The Tukey test indicates that the concentration of flavoglaucin by strains *E. rubrum* C47, *E. rubrum* NRRL 52, *E. rubrum* NRRL 76, and *E. repens* NRRL 40, and that of aspergin for the tree first strains, are significantly different among themselves and compared with the other strains studied. The differences in both compounds production among the other strains were not significant.

Production of flavoglaucin by *E. rubrum* C47 was maximal in CY20SM medium at 14 days of growth (25.1 μg/mg mycelium). Both *E. rubrum* NRRL 52 and *E. rubrum* NRRL 76 gave lower productions (11 μg/mg mycelium) which corresponds to 44% of that of *E. rubrum* C47. All the other strains tested gave amounts of flavoglaucin lower than 1.0 μg/mg mycelium at all the sampling times (Figure 6A). Surprisingly *E. cristatum* NRRL 4222, a strain with good acaricidal activity in mite tests did not produce flavoglaucin in solid medium CY20SM supporting that the miticidal action of this strain is produced by a different mechanism [18].

Production of aspergin was maximal (15 μg/mg mycelium) by *E. rubrum* NRRL 76 (a strain isolated from environment other than ham or cecina) grown in CY20SM medium for 14 days (Figure 6B), while *E. rubrum* C47 and *E. rubrum* NRRL52 produced amounts about 27% lower.

The production of both, flavoglaucin and aspergin, by strains grown in G25N medium were similar, but always lower than those of the strains grown in CY20SM medium with the only difference that aspergin was produced by *E. rubrum* C47 at higher levels (15 μg/mg mycelium) at 14 days of growth.

### 2.7. Formation and Release of Flavoglaucin to the Medium in Liquid Cultures

In order to determine the production of flavoglaucin and aspergin in liquid medium three strains, *E. rubrum* C47, and the collection strains *E. rubrum* NRRL 76 and *E. rubrum* NRRL 52 were grown in liquid CY20SM and G25N media for 192 h. Flavoglaucin and aspergin were determined by HPLC in extracts of the centrifuged mycelium and in the supernatant of the culture.

**Figure 7:**
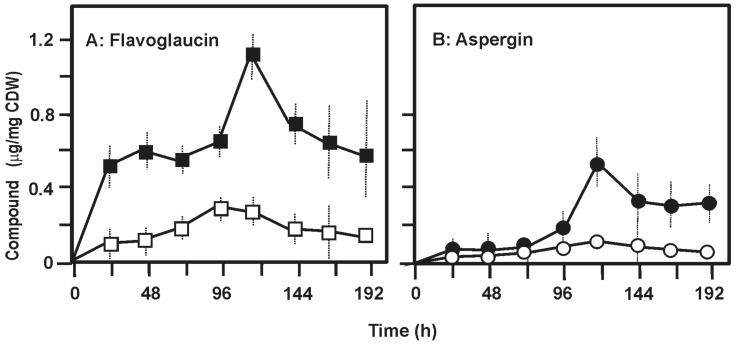
Production and release of flavoglaucin and aspergin in liquid cultures. Production of flavoglaucin (**A**) and aspergin (**B**) by cultures of *E. rubrum* C47 grown in liquid CY20SM medium. (**A**) Mycelium associated flavoglaucin (black squares) and flavoglaucin released to the supernatant (white squares). (**B**) Mycelium associated aspergin (black circles) and aspergin released to the supernatant (white circles). Data are the average of three cultures and standard deviations are shown by vertical dashed lines. Statistical analysis (*t*-student test) of flavoglaucin and aspergin concentration shows *t*-value > critical *t*-value, so that both compounds concentration is significantly different in mycelium and culture supernatant.

Production of flavoglaucin by *E. rubrum* C47 reached 1.12 μg/mg cell dry weight at 120 h of culture in liquid CY20SM medium (Figure 7A), well below the 25.1 μg/mg mycelium obtained in CY20SM solid cultures by the same strain. A surprising result was the finding that the maximal amount of flavoglaucin in the medium supernatant was in the order of 0.25 μg/mg CDW at 120 h, i.e., about 22% of the total flavoglaucin produced. Production of aspergin in liquid CY20SM reached a maximum value of 0.52 μg/mg CDW at 120 h and as occurred with flavoglaucin the levels of the compound in the broth were always below 25% of those found in the mycelium. These values of secretion were in the order of 10 to 20% in liquid medium G25N, and similar results were obtained with strains *E. rubrum* NRRL 76 and *E. rubrum* NRRL 52 confirming that both flavoglaucin and aspergin are poorly released into the culture broth (see discussion).

## 3. Discussion

Mite infestation of dry cured meat products constitute a serious problem because it deteriorates the quality of these products and, in addition, it produces allergies to the workers in the production factories. The infesting mites feed on mycelium and spores growing on the surface of ham and other meat products. We found that some of the fungi growing on the surface of ham and cecina produce a metabolite which have miticidal activity and is associated with abundant formation of cleistothecia [18]. Strains producing high levels of miticidal activity, particularly *E. rubrum* C47. were isolated and characterized to belong to the *Eurotium* genus (now reclassified *Aspergillus*/section *Aspergillus*) [24].

Mass spectrometry determination and NMR spectroscopy analysis have established that the acaricidal compound is a prenylated salicylic aldehyde with a C-7 alkyl chain and a molecular mass of 304. The structure of this compound, that carries a prenyl group coincides with that of flavoglaucin, a metabolite described as produced by some strains of the *Aspergillus glaucum* series [23,25,26]. The prenyl group probably is required for the biological activity since it has been shown that similar prenyl groups are essential for the bioactivity of other secondary metabolites [27].

A second product, aspergin (also named dehydroflavoglaucin), containing a double bond between carbons C8–C9 of the aliphatic chain (Figure 4) was also purified from cultures of *E. rubrum* C47. Interestingly this compound showed little or no miticidal activity, indicating that the saturated alkyl chain was essential for full miticidal activity. Noteworthy, the collection strain *E. rubrum* NRRL 76 that was not isolated from ham produces the highest level of aspergin in CY20SM medium as compared to *E. rubrum* C47; this suggests that there is a correlation between the origin of the *E. rubrum* strain and the production of aspergin versus flavoglaucin. It has been suggested that the prolonged growth of fungi in presence of mites has resulted in evolutionarily-acquired induction of the expression of genes encoding the miticidal compound [15,18].

The two metabolites characterized, flavoglaucin and aspergin, were produced simultaneously by the strains tested in distinct medium and culture conditions suggesting that both compounds are synthesized by the same biosynthetic pathway (Figure 5 and Figure 6). An important finding is that both flavoglaucin and aspergin remain cell-associated and are poorly released in the culture medium, which is consistent with the fact that these are hydrophobic compounds. The production of the miticidal compound is associated with cleistothecia [18] and it is known that many hydrophobic secondary metabolites are located in cleistothecia or asexual spores and are not efficiently released into the culture broth [28,29,30,31]

In the flavoglaucin molecules the C-7 lateral chain has been proposed to be part of a tetradecaketide synthesized by a high reducing polyketide synthase (HR-PKS) [32]. The initial seven carbon atoms of this tetradecaketide forms the C-7 alkyl group whereas the other seven carbons are cyclized to form the hydroxy-benzyl aldehyde ring of flavoglaucin. This PKS contains all six domains of a fully saturated polyketide synthase [32] including the enoyl reductase domain (ER) that converts the unsaturated intermediate into the fully reduced polyketide chain. The origin of the double bond in aspergin is still unclear. Either the HR-PKS fails to reduce one of the double bonds, that remains in aspergin, or double bonds may be introduced by a desaturase activity a posteriori. Fatty acid desaturases occur in filamentous fungi and are involved in the biosynthesis of unsaturated fatty acids that serve as regulator of the formation of asexual spores and sexual ascospores [33]. However, it is not clear if a specific desaturase is involved in flavoglaucin biosynthesis in *E. rubrum*, that may be encoded by a gene outside the flavoglaucin gene cluster.

So far, the main use of *E. rubrum* producing miticide is as starter culture in the biocontrol of mite infestation in ham [18] but there is the possibility that purified flavoglaucin could be used as an acaricidal agent in the control of mites in other infested systems, e.g., in the control of Varroa infections of honeybees, although previous studies on toxicity to the bees would be required. Flavoglaucin is an antioxidant compound formed during fermentation of the oriental food katsuobushi [34]. In addition to these biological activities flavoglaucin has been reported to be an inhibitor of tumor promoting by Epstein–Barr virus [27]; tumor promoting by this virus is a slow process and is reversed by flavoglaucin during the multiple steps of carcinogenesis. Early results on the toxicity of flavoglaucin indicated that this compound uncouples the oxidative phosphorylation chain in mitochondria, and increases the ATPase activity depleting the cellular ATP level in rat hepatocytes [35]. However, the effect on mites’ metabolism has not been studied yet.

## 4. Materials and Methods

### 4.1. Strains and Culture Conditions

*Eurotium rubrum* C47 was isolated in a screening program of fungi for acaricidal activity and classified taxonomically using molecular genetic tools [18]. *E. rubrum* C47 was grown in (1) solid CY20SM medium (beef extract 5 g; sucrose 200 g; K_2_HPO_4_ 1 g; NaCl 5 g; KCl 0.5 g; NaNO_3_ 3 g; MgSO_4_·7H_2_O 0.5 g; FeSO_4_.7H_2_O 0.01 g; pH 5.2; agar 15 g), and (2) CY20S medium (yeast extract 5 g; sucrose 200 g; K_2_HPO_4_ 1 g; KCl 0.5 g; NaNO_3_ 3 g; MgSO_4_.7H_2_O 0.5 g; FeSO_4_·7H_2_O 0.01 g; pH 5.2; agar 15 g). In addition, other media, (G25N, PDAM, MEAM, PWM, PW2M, SDAM and RTAM), detailed in Table S1, were used to optimize the acaricidal compounds production.

To produce the acaricidal compounds in solid medium 10^7^ conidia of *E. rubrum* C47, conserved in 40% glycerol, were applied to a sterile filter paper disc of the same diameter of plates containing solid CY20S medium. The plates were incubated at 28 °C in humid atmosphere. To obtain the acaricidal compound in liquid medium *E. rubrum* was grown for 48 h at 28 °C and 220 rpm in 500 mL flasks containing 100 mL of CY20S liquid medium. Then 10 mL of this pre-culture were used to inoculate flasks containing the same medium and culture conditions.

In solid cultures the mycelium was collected from a disk of filter paper adhered to the solid medium and weighted. Growth was quantified in liquid cultures as cell dry weight (CDW). The mycelium obtained by centrifuging 5 mL of liquid culture was washed three times with saline solution and dried at 80 °C until constant weight.

Food substrate for the growth of mites. The mites were fed with Dry Yeast Substrate (DYS) containing wheat germ 75% and beer yeast 25%.

### 4.2. Determination of the Acaricidal Activity of Extracts and Liquid Culture Samples

*Tyrolichus casei* was used routinely to test the acaricidal activity in extracts. Two additional mites, *Tyrophagus putrescentiae* and *Tyrophagus longior*, were used to confirm the results obtained with *T. casei* [18]; these results are not described here in detail since the miticidal effect was similar for the three mites. The mites were kept at 25 °C on DYS, in 50 mm diameter plates, placed in a closed glass beaker on top of a water filled vessel to maintain 80 to 90% humidity.

The acaricidal activity of the samples along the purification process was determined by evaporation of the sample to dryness, resuspension in 100 μL ethanol and application of the solution to 100 mg of DYS. The solvent in the food was vacuum eliminated and the plates were kept for 24 h at 4 °C in humid atmosphere. Then a fixed number of mites (usually 20 individuals of both sexes) were deposited on the plates. The assay was maintained for 15 days at 25 °C and 90% humidity with periodical counting and observation of the mites with a binocular loupe. All experiments were repeated three times.

### 4.3. Statistical Studies

Results obtained in this work were submitted to an analysis of variance (ANOVA) to compare averages and determinate if some of them are significantly different from the rest. When *p*-value is less than 0.05 (95% confidence level) that means at least one group is significantly different; we make a Tukey’s range test (honestly significant difference “HSD”) to determine which condition of all them is significantly different. The *t*-student test has been used to determine whether there is a significant difference between averages of two samples.

### 4.4. Purification of the Acaricidal Compounds

Obtention of total extracts from the fungal mycelium. The mycelium of 40 plates of *E. rubrum* C47 grown for 14 days on filter paper placed on top of the solid media plates was collected (24 g), extracted four times with 25 mL ethyl acetate, shaken in a Vortex for 5 min, and centrifuged for 10 min at 4400 rpm. The four extracts were mixed, concentrated to dryness and dissolved in dichloromethane-methanol (4:1 *v*/*v*).

Fractionation by Silica Gel chromatography. Extracts obtained in the previous step were applied to a 800 × 40 mm glass column filled with Silica Gel (Sigma Chem. Co., St Louis, MO, USA) equilibrated with dichloromethane. Step-wise elution was performed with 1.8 l of dichloromethane with a flow of 15 mL/min (Fraction I), then 1.8 l of dichoromethane:methanol (1:1) with a flow of 12.5 mL/min (Fraction II) and finally 1.8 L of dichloromethane-methanol (1:9) with a flow of 12.5 mL/min (Fraction III). Fractions I, II and III were separately concentrated to dryness; the entire solid residue of each fraction was dissolved in 25 mL of acetone and the acaricidal activity was determined in each extract.

Gel Filtration Chromatography in Biogel S-X3. Two ml of the fraction with acaricidal activity obtained after Silica Gel fractionation were applied to a 120 × 1.5 cm Bio-Beads S-X3 (Bio-Rad, Hercules, CA, USA) column previously equilibrated with acetone. The sample was eluted with pure acetone at a flow of 1 mL/min taking fractions every 5 min. The fractions were tested for acaricidal activity and the active fractions were combined, concentrated in the minimal volume of acetonitrile, centrifuged at 132,000 rpm for 15 min to remove solid residues and applied to an HPLC semipreparative column.

Semipreparative HPLC. Samples with acaricidal activity obtained from gel filtration in Biogel S-X3, were dried, dissolved in acetonitrile and applied to a reverse phase LiChrospher 100 RP-18 column (250 × 10 mm) with a pore size of 10 μm. The chromatography was performed in a Waters TM 650E chromatograph equipped with a pump model 600 and a fix wavelength detector model 486 set at 260 nm. The elution was carried out at a flow of 7.5 mL/min using as mobile phase water (solvent A) and acetonitrile (solvent B) and the following lineal gradients: Time 0 min 50% B; time 8–12 min, 70% B; time 16–22 min, 90% B; time 25–35 min, 50% B. Samples with the different peaks were collected, lyophilized, dissolved in ethanol and tested for acaricidal activity.

Analytical HPLC. The samples with acaricidal activity obtained by semipreparative HPLC were dried, dissolved in acetonitrile, centrifuged at 13.200 rpm for 15 min to remove solid residues and applied to a LiChrospher 100 RP-18 column (250 × 4 mm) with a pore size of 5 μm. The chromatography was performed using a Shimadzu model 10AD-VP equipped with a photodiode detector model SPD-M10AVP. The elution was carried out with a flow of 0.7 mL/min using water (solvent A) and acetonitrile (solvent B) and the follow lineal gradients: Time 0 30% B, time 2 min 80% B; time 23 min 90% B; time 25 to 30 min 50% B.

### 4.5. Thin Layer Chromatography

Samples (10 μL) from the different purification steps were applied to Silica-gel 60 chromatosheets (Merck, Darmstadt, Germany). The chromatosheets were placed in a chamber saturated with the mobile phase (benzene:dichloromethane 4:1) and the chromatography was developed. The chromatosheets were dried and the compounds were visualized with a spray of developing solution (phosphomolybdic acid 20% in ethanol) and heating at 120 °C for 30 s.

### 4.6. Structure Elucidation of Compounds by Mass Spectrometry and Nuclear Magnetic Resonance

MS data were performed with a HP1100 liquid chromatograph equipped with a gradient pump, a photodiode-array detector and a single quadrupole mass spectrometer (Agilent Technologies, Santa Clara, CA, USA), using both, ESI (+) and (−) and APCI (+) and (−) ionization sources. ^1^H-NMR and ^13^C-NMR spectra (Figure 1) were acquired on a Varian Mercury 400 NMR spectrometer (400 MHz for ^1^H, 100 MHz for ^13^C). Chemical shifts are reported in ppm using residual CDCl_3_ (δ 7.26 ppm for ^1^H and 77.0 ppm for ^13^C) as an internal reference. COSY, gHMQC and gHMBC experiments were carried out using an inverse resonance probe and standard pulse sequences.

### 4.7. Quantification of Flavoglaucin and Aspergin

Both compounds were purified to homogeneity by HPLC chromatography. One milligram of each compound was dissolved in 1 mL of dichloromethane-methanol (4:1 *v*/*v*) and serial dilution samples were analyzed by HPLC to get a plot correlating amounts of the compound and absorption at 390 nm.

## 5. Conclusions

We have purified to homogeneity and characterized a potent acaricide compound produced by cultures of *Eurotium rubrum* C47 isolated from the surface of cured ham. Using mass spectrometry and NMR analysis we identified two compounds as prenylated salicylaldehydes with a C7 alkyl side chain. One of them, identified as flavoglaucin, has a potent miticidal activity. The second compound, differing in the presence of a double bond in the lateral chain, was identified as aspergin and has no significant miticidal activity. Both compounds remain largely cell associated and are not released in the culture broth. In summary, cultures of *E. rubrum* C47 are important biocontrol tools for the removal of ham infesting mites.

## Figures and Tables

**Table 1:** ^1^H NMR (400 MHz) and ^13^C NMR (100 MHz) spectral data for Compounds 9b and 9c in CDCl_3_.

C/H No	Flavoglaucin		Aspergin	
	^13^C ^a^	^1^H ^a,b,c^	^13^C ^a^	^1^H ^a,b,c^
1	195.8	10.25 s, 1H	196.5	10.09 s, 1H
2	117.5		117.3	
3	128.7		130.6	
4	145.2		145.0	
5	125.9	6.88 s, 1H	125.3	7.02 s, 1H
6	128.7		128.7	
7	156.0	11.92 s, OH	155.4	11.73 s, OH
8	24.2	2.88 t, 2H (*J* = 8.0)	120.3	6.47 d, 1H (*J* = 14.0)
9	32.2	1.56 m, 2H	142.9	6.00 dt, 1H (*J* = 14.0, 6.8)
10	29.4	1.30 m, 2H	33.7	3.34 m, 2H
11	29.8	1.38 m, 2H	31.7	1.35 m, 2H
12	32.0	1.26 m, 2H	28.9	1.50 m, 2H
13	22.8	1.28 m, 2H	22.7	1.36 m, 2H
14	14.3	0.87 t, 3H (*J* = 7.0)	14.2	0.89 t, 3H (*J* = 6.9)
15	27.2	3.29 d, 2H (*J* = 7.2)	27.4	3.31 d, 2H (*J* = 7.3)
16	121.4	5.58 m, 1H	121.2	5.58 m, 1H
17	134.1		134.2	
18	18.0	1.70 s, 3H	18.0	1.70 s, 3H
19	26.4	1.76 s, 3H	26.4	1.76 s, 3H

^a^ Chemical shifts (δ) in ppm; ^b^ s: singlet, d: doublet, t: triplet, m: multiplet; ^c^ Coupling constants (*J*) in Hz.

## Supplementary Materials

The following are available online at https://www.mdpi.com/2079-6382/9/12/881/s1. Figure S1: Acaricidal activity in extracts, Figure S2: Absorption spectra of fractions 9b and 9c, Table S1: Culture media used to optimize flavoglaucin production.
