# Supplementary material for: Purification and Chemical Characterization of a Potent Acaricide and a Closely Related Inactive Metabolite Produced by Eurotium rubrum C47
Source: Antibiotics (Basel). 2020 Dec 9;9(12):881. doi: 10.3390/antibiotics9120881 (PMC7763031; doi:10.3390/antibiotics9120881)
Supplement: Supplementary file 1 [file antibiotics-09-00881-s001.pdf]

antibiotics

MDPI

Supplementary Material

Table S1. Culture media used to optimize flavoglaucin production.

Name	Composition in grams/liter
**G25N**	Yeast extract, 3,7; glycerol, 315; KCl, 0.03; $NaNO_3$, 0.22; K_2HPO_4, 0.75; $MgSO_4.7H_2O$, 0.03; $FeSO_4.7H_2O$, 0.00075; pH 7.0; agar 15.
**PDAM**	Potato infusion, 4; glucose, 20; sucrose, 200; pH 5.6; agar, 20.
**MEAM**	Malt extract, 15; peptone, 15; NaCl, 10; glucose, 20; sucrose, 200; pH 5.6; agar, 15
**PWM** (modified Power medium)	Bacto peptone, 5; corn steep solids, 0,5; lactose, 30; NaCl, 4; KCl, 52.2; K_2HPO_4, 0.5; KH_2PO_4, 0.060; $MgSO_4.7H_2O$, 0.55; $FeCl_3.6H_2O$ 0.003; $CuSO_4.5H_2O$, 0.001; $NaNO_3$, 2; $FeSO_4$. $7H_2O$, 0.010; sucrose, 200; pH 6.75; agar 25.
**PW2M** (As PWM medium with the following modifica-tions/additions)	Corn steep solids, 1; lactose, 10; KCl, 104; $MgSO_4.7H_2O$, 1.
**SDAM** (modified Sabouraud medium)	Neopeptone Difco, 10; glucose, 40; sucrose, 200; pH 5.6; agar, 20
**RTAM**	Tartaric acid, 4; $NaHCO_3$, 0.6; $(NH_4)_2HPO_4$, 0.6; $(NH_4)_2SO_4.7H_2O$ 0.25; $Fe(NH_4)_2(SO_4)_2$. 6 H_2O 0.070; Zn SO_4. 7 H_2O, 0.070; MgSO4. 7H2O, 0.4; sucrose, 200; pH 5.2; agar, 15

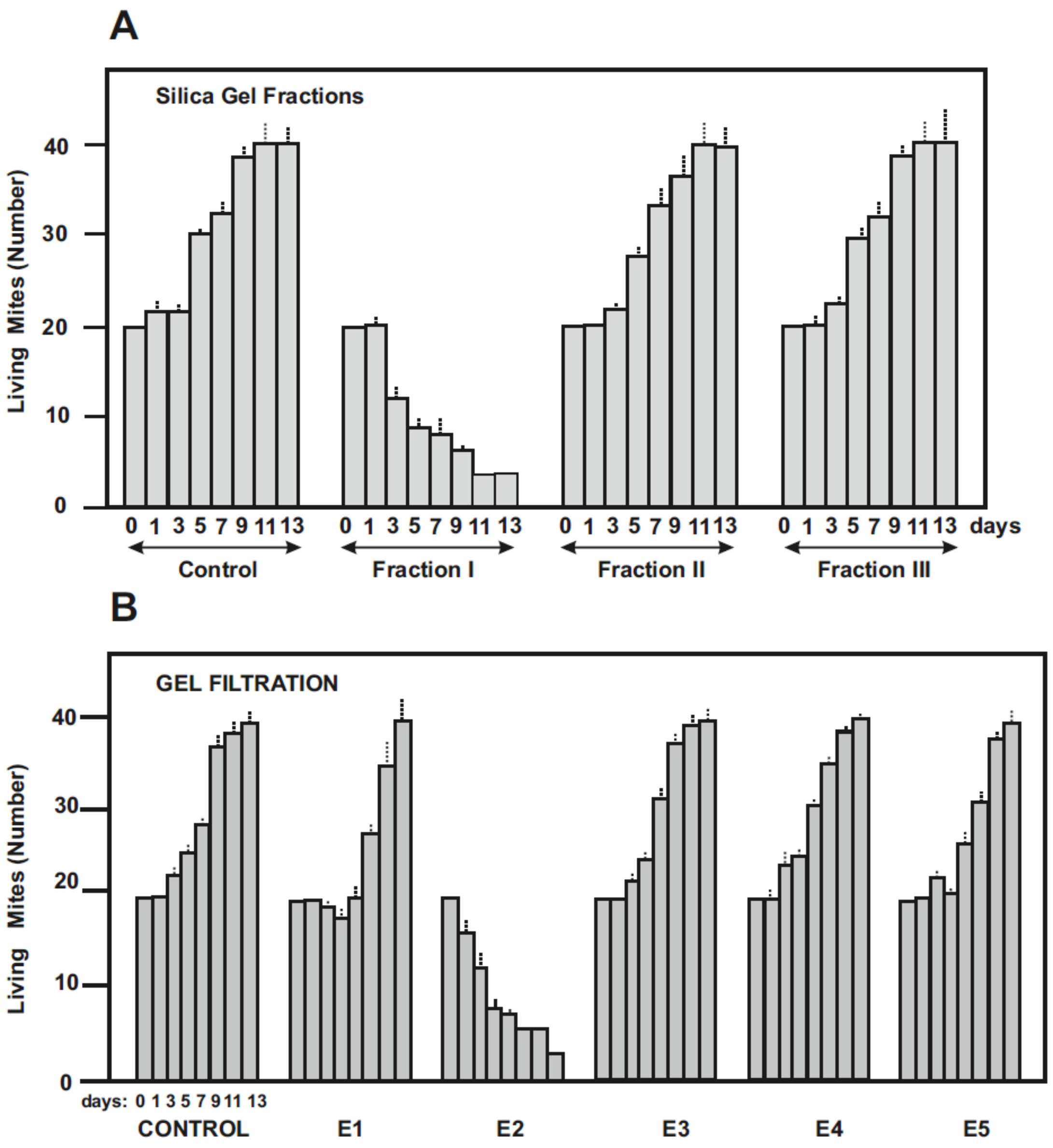

Figure S1. Purification of the acaricidal compound by Silica Gel chromatography and gel filtration in Bio Gel S-X3. **A**) Acaricidal activity measured at 0, 1, 3, 5, 7, 9, 11 and 13 days in 50 mm Petri dishes containing 100 mg of DYS mite food supplemented with 100 ml ethanol (Control), or 100 ml of ethanolic solutions of vacuum dried fractions I, II and III. Statistical analysis (ANOVA) of living mite numbers in the three silica gel fractions and the control sample indicates a p-value<0.05. The Tukey test shows that the difference between fraction I and the other samples is significant. However, the differences between the control and fractions II and III is not significant. **B**) Acaricidal activity measured at 0, 1, 3, 5, 7, 9, 11 and 13 days of DYS mite food supplemented with 100 ml ethanol (Control), or 100 ml of ethanolic solutions of vacuum dried fractions E1, E2, E3, E4, E5 obtained by chromatography in Bio Gel S-X3 (see Materials and Methods for details). ANOVA analysis of living mite number in six samples analysed indicates a p-value<0.05. At 7 days the Tukey test shows a significant difference between E1 and E2 and with the other samples (E3, E4, E5 and control). The differences among the other samples is not significant. At 13 days exist a significant difference in E2 in relation to all the other samples.

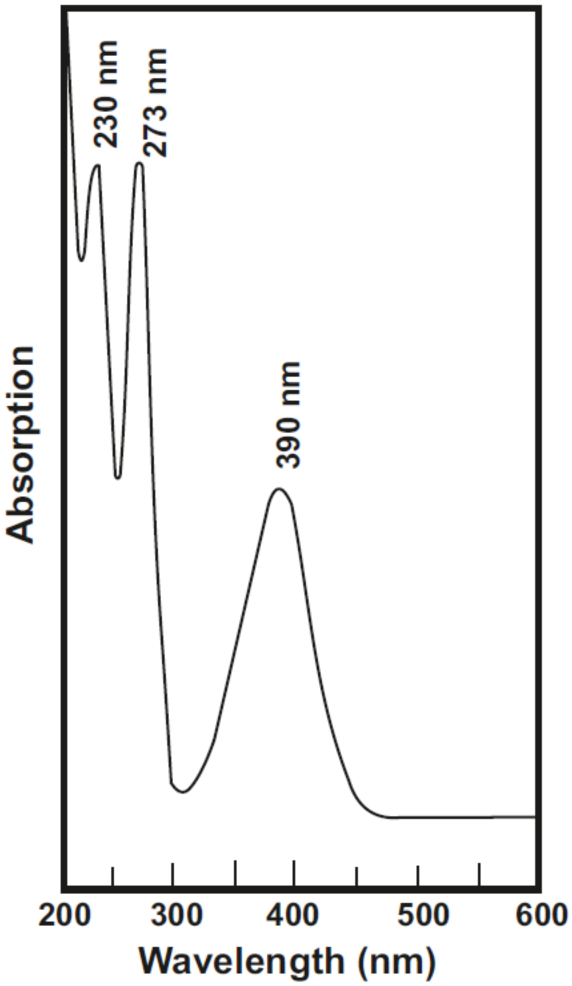

Figure S2. Absortion spectra of fractions 9b and 9c obtained by analytic HPLC. The scanning between 200 nm and 600 nm showed in both fractions the same maximal absortion peaks at 230, 273 and 390 nm.

Publisher's Note: MDPI stays neutral with regard to jurisdictional claims in published maps and institutional affiliations.

© 2020 by the authors. Licensee MDPI, Basel, Switzerland. This article is an open access article distributed under the terms and conditions of the Creative Commons Attribution (CC BY) license (http://creativecommons.org/licenses/by/4.0/).
